# Angry back

**DOI:** 10.1002/ski2.428

**Published:** 2024-07-25

**Authors:** Rosanne Ottevanger, Roel E. Genders

**Affiliations:** ^1^ Department of Dermatology Leiden University Medical Center Leiden Netherlands

## Abstract

A healthy 16‐year‐old boy presented with a rash and painful back. This occurred minutes after an acrobatic act in the swimming pool where he landed flat on his back. The lesions were painful. He was diagnosed with pressure urticaria.

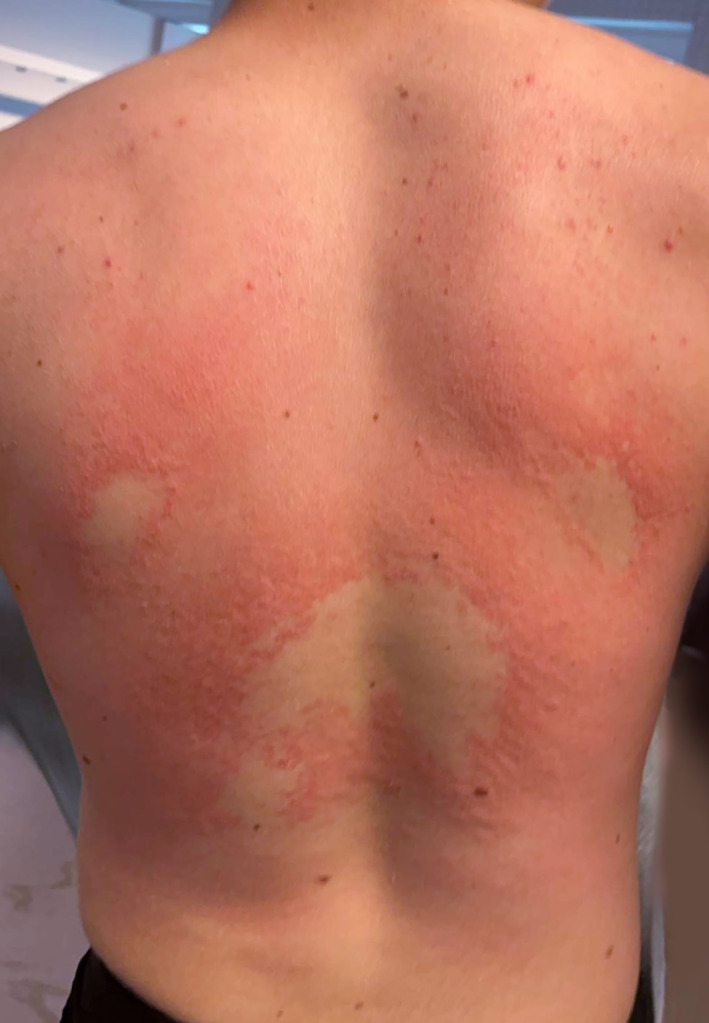

A healthy 16‐year‐old boy presented with a rash and painful back. This occurred minutes after an acrobatic act in the swimming pool where he landed flat on his back (Figure 1 and Video S1). Examination showed diffuse confluent raised erythema on his back, with some symmetrical non‐affected skin. He reported to be feeling a little feverish but reported no chills. The lesions were painful. The swelling spontaneously resolved within a few hours after the incident. His back remained painful with only minimal spotted erythema that was resolved the next day. The lesions were diagnosed as pressure urticaria. Dermographism was positive. Pressure urticaria are uncommon, but probably not rare. They occur immediately after a pressure stimulus or sometimes after a delay of 4–6 h. The lesions last for 8–72 h. The activation of mast cells, triggered by pressure, play an important role in the pathogenesis. It can be treated with systemic antihistamines alongside analgesia.

**FIGURE 1:**
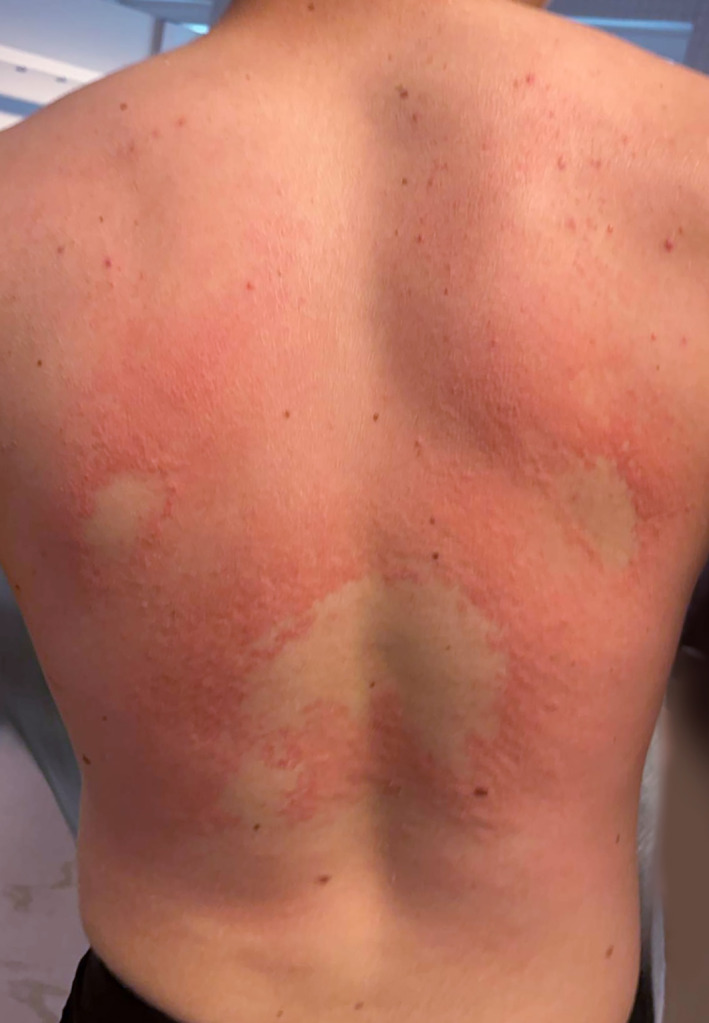
Diffuse confluent raised erythema on his back, with some symmetrical non affected skin minutes after the fall.

## CONFLICT OF INTEREST STATEMENT

The authors declare no conflicts of interest.

## AUTHOR CONTRIBUTIONS

**Rosanne Ottevanger**: Conceptualization (equal); writing—original draft (equal). **Roel E. Genders**: Conceptualization (equal); supervision (equal); writing—review and editing (equal).

## ETHICS STATEMENT

Not applicable.

## PATIENT CONSENT

Written patient consent for publication was obtained.

## Supporting information

Videos S1

## Data Availability

The data underlying this article will be shared on reasonable request to the corresponding author.

